# Characteristics of outdoor falls among older people: a qualitative study

**DOI:** 10.1186/1471-2318-13-125

**Published:** 2013-11-18

**Authors:** Samuel R Nyman, Claire Ballinger, Judith E Phillips, Rita Newton

**Affiliations:** 1 Bournemouth University Dementia Institute and Psychology Research Centre, School of Design, Engineering & Computing, Bournemouth University, Poole House, Talbot Campus, Poole, Dorset BH12 5BB, UK; 2 Research Design Service South Central, Faculty of Medicine, University of Southampton, Tremona Road, Southampton SO16 6YD, UK; 3 Centre for Innovative Ageing, Swansea University, Singleton Park, Swansea SA2 8PP, UK; 4 SURFACE Inclusive Design Research Centre, School of the Built Environment, The University of Salford, The Crescent, Salford M5 4WT, UK

**Keywords:** Accidental falls, Outdoors, Older people, Environment and public health, Fear of falls, Qualitative research

## Abstract

**Background:**

Falls are a major threat to older people’s health and wellbeing. Approximately half of falls occur in outdoor environments but little is known about the circumstances in which they occur. We conducted a qualitative study to explore older people’s experiences of outdoor falls to develop understanding of how they may be prevented.

**Methods:**

We conducted nine focus groups across the UK (England, Wales, and Scotland). Our sample was from urban and rural settings and different environmental landscapes. Participants were aged 65+ and had at least one outdoor fall in the past year. We analysed the data using framework and content analyses.

**Results:**

Forty-four adults aged 65 – 92 took part and reported their experience of 88 outdoor falls. Outdoor falls occurred in a variety of contexts, though reports suggested the following scenarios may have been more frequent: when crossing a road, in a familiar area, when bystanders were around, and with an unreported or unknown attribution. Most frequently, falls resulted in either minor or moderate injury, feeling embarrassed at the time of the fall, and anxiety about falling again. Ten falls resulted in fracture, but no strong pattern emerged in regard to the contexts of these falls. Anxiety about falling again appeared more prevalent among those that fell in urban settings and who made more visits into their neighbourhood in a typical week.

**Conclusions:**

This exploratory study has highlighted several aspects of the outdoor environment that may represent risk factors for outdoor falls and associated fear of falling. Health professionals are recommended to consider outdoor environments as well as the home setting when working to prevent falls and increase mobility among older people.

## Background

Falls are globally recognised as a major threat to the health and wellbeing of older people [1]. Falls in adults aged 65+ account for over 50% of injury-related hospital admissions and 40% of all injury deaths, are costly for health services to treat, and may result in fear of falling that prevents older people from getting outdoors [1-4]. Current evidence suggests that both falls and risk of falls are prevented in community settings by individuals regularly carrying out specific physical activities and improving the safety of their homes [5]. However, despite progress in research there remain at least two gaps in the current falls literature. First, at the expense of knowledge on environmental risk factors for falls, researchers have concentrated on identifying and addressing individual risk factors such as prescribing exercise to address deficits in strength and balance [6,7]. Although there is some evidence for the effectiveness of home safety interventions to prevent falls (and with greater effect among high-risk groups for example, through reducing tripping hazards) [5,8], the evidence comes from fewer than seven trials [5,8] and does not yet translate to a reduction in fall-related injury [9]. Consequently, there is a lack of robust evidence for environmental risk modification.

Second, outdoor falls have been neglected, as most research has focused on falls occurring in the home or hospital environment. Outdoor falls are frequent; approximately half of falls among adults aged 65+ occur in outdoor environments [10-13]. Outdoor falls also have risk factors that are distinct from indoor falls and are linked with risk exposure [14]: those who fall outdoors are more likely to be male, younger, and more physically active and healthy (are more independent in activities of daily living and have a faster gait speed) [10-13,15-20]. However, despite the high frequency of outdoor falls and the distinct emergent set of risk factors described, current definitions of falls do not distinguish between indoor and outdoor falls [21]. Thus, current guidelines on fall prevention recommend modification of the home environment as a component of a multifactorial intervention, but include no specific recommendations for the prevention of outdoor falls [7] and no guidance to local authorities and other bodies responsible for maintaining public areas. Consequently, beyond sociodemographic risk factors for outdoor falls, little is known of the contexts in which outdoor falls occur, how features of the external environment can present as risk factors for outdoor falls, and which outdoor falls are more likely to lead to injury and/or fear of falling. The current qualitative study aimed to begin to fill the above two gaps in the falls literature by examining in detail older people’s retrospective accounts of outdoor falls. The aims of this research study were therefore to explore the contextual factors associated with outdoor falls, and explore the interrelationship of these factors with injury and fear of falling.

## Methods

### Design

We conducted focus groups: semi-structured discussion groups of around three to seven people that are facilitated to comment on a particular topic [22,23]. Although similar to interviews, the group context of focus groups make them ideal for providing a breadth of experiences and views on a topic [23], and for gaining access to participants’ collective understandings [24]. While data obtained from focus groups may not generalise to a national population, such an exploratory study is important in order to highlight areas to be emphasised in future large-scale quantitative work. Focus groups were an appropriate tool to collect exploratory, qualitative data on this subject given the paucity of research in this area and our aim to ascertain several older people’s views on the research question. They were also ideal as it afforded ease of recruitment across the United Kingdom, as detailed below. The focus group schedule was orientated around our main research aims and six sub-research questions (see Additional file 1 for the focus group schedule). This paper details the findings in relation to older people’s experiences of outdoor falls.

### Participants

A purposive sampling strategy was employed in order to identify older people likely to offer a diversity of views relating to the research topic. Given that this study concerned older people’s experiences of falls in outdoor environments, we were interested in recruiting participants from different geographical contexts. For this purpose, focus groups were conducted in both urban and rural areas in five different locations across the United Kingdom (UK), representing north (Scotland), west (Wales), south, east, and central parts of the UK (three sites in England) (see Table 1). In addition to purposive sampling at group level, we employed quota sampling in an attempt to recruit participants of both genders, and relatively younger (aged 65–70 years) and older (aged >80 years). This strategy was largely successful, although we experienced difficulty in recruiting men into the focus groups. The inclusion criteria for participation in the study were: aged 65 and above, able and willing to reflect on their own and others’ experiences of outdoor falls, experienced an outdoor fall in the past 12 months, and currently going outdoors as a pedestrian at least once every week.

**Table 1:** Characteristics of focus group participants

**Location**	**Group no.**	***n***	**Living environment**		**Living arrangement**		**Independence with everyday tasks at home**		**Outings per week**	**Highest level of education**		**Gender**		**Age (years)**	
			**Urban**	**Rural**	**Alone**	**With others**	**Independent**	**Receive care**^**1**^	**Range**	**Mandatory school education**^**2**^	**Further/higher education**^**3**^	**M**	**F**	***M***	**Range**
Wales	1	6	6	0	1	5	6	0	1 - 14	3	3	3	3	78.17	69 - 87
	2	4	2	2	2	2	3	1	2 - 7	0	4	1	3	70.25	69 - 71
	3	6	0	6	3	3	6	0	1 - 7	2	4	1	5	80.00	75 - 84
England	4	5	5	0	4	1	5	0	3 - 7	2	3	1	4	76.40	69 - 87
	5	7	7	0	4	3	6	1	4 - 7	2	5	0	7	78.71	69 - 91
	6	4	0	4	3	1	3	1	2 - 5	3	1	0	4	84.50	82 - 88
	7	3	0	3	1	2	3	0	2 - 5	3	0	0	3	70.67	65 - 79
Scotland	8	4	4	0	1	3	3	1	1 - 7	4	0	1	3	80.75	70 - 92
	9	5	3	2	5	0	5	0	3 - 6	5	0	0	5	79.60	74 - 85
Total		44	27	17	24	20	40	4		24^4^	20^5^	7	37		

^1^Participants were asked how they manage with tasks in their home on a day-to-day basis, of which three reported that they received carer support, one received tele-care/tele-health, and the remainder stated they were independent.

^2^Participants who had received education at primary school level (up to 12 years of age) or secondary school level (up to 16 years of age), with qualifications at secondary school usually in the form of General Certificates of Secondary Education.

^3^Participants, who in addition to school education had received further education or obtained a professional qualification (e.g. to perform a trade), or received education at a higher education college or university (e.g. Bachelor of Science or Doctor of Philosophy).

^4^Four at primary level, 20 at secondary level.

^5^Two at higher education college, 5 at university, and 13 obtained further education or a professional qualification.

Between March and June 2012 we conducted nine focus groups with 3–7 (median = 5) participants per group, and a total of 44 participants. Participants were recruited through voluntary and private organisations, and local authorities. Participants were identified for focus groups either through the use of existing contacts or through making contact with venues that older people frequent such as day centres and social clubs. Participants were not screened for long-term health issues, however, a group of older people with low vision was recruited from a voluntary organisation as they represent those at an increased risk of outdoor falls [14,25]. In addition, we recruited users of a post-fall service provided by the National Health Service (NHS) as they tend to represent frailer older people who have experienced an injurious fall requiring medical treatment. NHS patients were identified by the direct care team either at initial home visits or at the beginning of exercise classes. For each focus group, those who expressed an interest in the study were sent a letter with an information sheet by the focus group facilitator, and if they met the entry criteria and agreed to participate, were recruited into a focus group that was arranged locally at a mutually convenient time.

### Ethics

Before recruitment commenced, approval was granted by the Research Ethics Committee of Bournemouth University. In addition, for the focus group conducted with NHS patients in Scotland, prior approval was provided by the local NHS research ethics committee (West of Scotland Research Ethics Service reference 12/WS/0101) and Research and Development office (NHS Greater Glasgow and Clyde Research and Development Office reference GN12OR164). Each participant was provided with refreshments and reimbursement of their travel expenses. To ensure anonymity in the data analysis, the identity of participants was concealed in the verbatim transcriptions of the focus groups by omitting participants’ names and other potentially identifiable characteristics (e.g. road name for current address).

### Procedure

The focus group venues comprised meeting rooms in universities, local authorities, voluntary organisations, and church and village halls. Each focus group was audio recorded, transcribed verbatim, and lasted on average 80 minutes (range = 46–107 minutes). The facilitator was accompanied by at least one assistant who helped with the practical running of the groups (e.g. collecting consent forms) and made notes on the order of speakers to help with audio transcription. At the beginning of each focus group, participants were reminded of the purpose of the focus group, given a copy of the information sheet again, and informed consent forms were signed. Ground rules for participating in the focus groups were discussed, including respect for others’ views, taking turns in speaking to aid audio recording, and commitment to confidentiality of the content of the focus group discussion. Focus group questions were made available to each participant on cards in large font, one question to a page, and the facilitator asked participants to comment on each question. The group were encouraged to interact with each other, with the facilitator intervening solely to keep the discussions on topic and to encourage quieter members of the group to speak. At the end of the discussion, participants were reminded of the ground rules for participation and in particular confidentiality of the content of the discussion. Participants then completed a questionnaire to provide demographic details, and each participant received a debrief form with contact details of the research team. Focus groups were led by one of two facilitators who were both academic researchers, female, aged in their fortes or fifties, and not responsible for clinical care of older people or maintenance of built environments. The focus groups were analysed by SRN and CB, academic researchers with a background in psychology and occupational therapy/social sciences.

### Analysis

The inclusion criteria for outdoor falls were that the fall occurred within the past two years in an outdoor space - whether built environments (such as the local shopping street) or more natural environments (such as parks) - either in the UK or overseas e.g. while on holiday. In determining frequencies, we included falls, injurious falls, and near-falls given that our focus was on risk factors for falls. A near fall was defined as an occasion on which an individual felt that they were going to fall but did not actually fall [26] (e.g. if able to break their fall by grabbing onto railings or had their fall broken by falling against a wall). Due to the volume of qualitative data gathered, framework analysis was initially employed to ensure a systematic and comprehensive approach [27]. It is a method that has been used before in the falls literature (e.g. [28]) and is highly suited to applied health research [27]. SRN conducted a framework analysis [29] using a comprehensive spreadsheet in Microsoft Office Excel ©2007, with each iteration of coding sorted by worksheets. The sub-research questions for the study were used as a deductive framework to organise the data with columns as broad codes, and each fall event populating an individual row (see Additional file 1 for the sub-research questions). The analysis entailed three iterations of coding, reliability checking and refinement of the coding, and comparisons between codes (as detailed in the results section) (see Table 2 for details). Content analysis was then used to determine the frequencies of events of interest [30], for example, which features of the physical environment were most frequently reported in the experiences of outdoor falls. Quotations were selected from across the sample (different participants and different focus groups) to collectively illustrate the range of different outdoor falls experienced. Demographic details of participants were descriptively analysed using Microsoft Office Excel ©2007.

**Table 2:** Framework analysis of focus group data

**Task**	**How performed**	**Coding structure**
First iteration of coding		
Each transcript was coded for the experience of outdoor falls recounted by participants, with each outdoor fall initially broadly coded by context and impact.	Each fall event was sorted by participant, the focus group they attended, and had quotations pasted into cells to justify each code. Each cell was populated with descriptions that captured the substantive content of the transcript excerpt.	Context of the outdoor fall was initially split into four broad codes:
		1) Characteristics of the environment (such as weather, lighting, incline, etc.)
		2) Social context (such as alone, talking with another, etc.)
		3) Familiarity (in familiar/unfamiliar area)
		4) Attribution (perceived cause of the fall)
		Impact of the fall was initially split into three broad codes:
		1) Physical injury
		2) Emotional reaction
		3) Anxiety about falling again
Indexing.	The above broad framework was systematically applied to all the transcripts.	
Consensus on number of outdoor falls recounted and refinement of inclusion criteria.	Independent coding by two researchers followed by discussion. The above coding was used to reach consensus on which falls occurred outdoors and were therefore to be included in the remainder of the analysis.	
Second iteration of coding		
The initial coding was then subcoded to capture the multiple variations of contexts and impacts of outdoor falls.	Each cell was refined to not only capture the substantive content but the dimensions of the transcript excerpts according to the sub-codes.	
Indexing.	New subcodes were generated as analysis progressed from the first to the final transcript, along with refinement of existing subcodes (e.g. splitting the subcode “season” into the four separate seasons of the year, and collapsing two similar subcodes into one subcode).	
Summaries were produced from the analysis that captured the total number of falls that occurred, and the patterns that emerged from the multiple sub-codes (known as charting).	The frequency of subcodes that emerged across participants was noted.	
Third iteration of coding		
While producing the summaries noted above, the sub-codes were reviewed and refined.		
Reliability checking and refinement		
Independent reliability checking, with particular attention to a code that was deemed to warrant further refinement: physical injury from falls (under the broad code of impact of falls).	The overall coding framework was checked by another researcher (CB). Each item coded under the broad code of impact of falls was then checked by the researcher (CB) and a falls practitioner (physiotherapist and lead for a local hospital-based falls team).	In refining the codes, we arrived at four broad codes:
		1) Features of the physical environment
		2) Features of the social environment (including familiarity of the location of the fall)
		3) Attributions
		4) Impact (physical injury with emotional response including anxiety about falling)
Refinement of coding of physical injury from outdoor falls.	We employed a coding framework used previously [13,31].	In using this framework we arrived at four codes for physical injury:
		1) No injury
		2) Untreated injury (minor injury that did not receive medical treatment)
		3) Treated injury (injury that received medical treatment, such as presenting to a family physician, hospital, or accident and emergency ward)
		4) Fractures (major injury or multiple fractures requiring hospital treatment)
Comparisons		
We made comparisons between the codes that emerged as most prevalent amongst the sample (known as mapping and interpretation).	The context of outdoor falls was compared with the impact of outdoor falls in terms of (a) physical injury and (b) anxiety about falling again.	

## Results

Forty-four adults aged 65 – 92 (mean age = 78) took part in the focus groups (see Table 1). The majority of participants were female (n = 37), considered themselves independent with everyday tasks at home (n = 40), and just over half lived in an urban area (n = 27) and lived alone (n = 24). Half the participants had received mandatory school education and half had received further/higher education. Of the 44 participants, 37 wore spectacles, 13 used a walking aid (walking stick or rollator), and 3 used a hearing aid. While nine reported no long-term health conditions, the remaining participants mainly reported one (n = 23) or two (n = 11) conditions, which were predominantly the following: arthritis (n = 8), hypertension (n = 7), osteoporosis (n = 5), diabetes (n = 4), asthma (n = 3), and other cardiovascular (n = 4) or respiratory related (n = 2) diseases. In addition to the four participants recruited from a visual impairment voluntary organisation, five others reported difficulty with their vision including three who were partially sighted. Across the nine focus groups, 124 falls were described (range 7 – 21 (mean = 14) per focus group). Of these falls, 88 occurred in outdoor environments in the past two years (range 5 – 17 (mean = 10) per focus group) and were included in our analysis. The mean number of outdoor falls per participant was 2 (range 1 – 7) (see Table 3). Fifty-three outdoor falls were reported by 24 participants from urban settings, and 35 outdoor falls were described by 17 participants from rural settings.

**Table 3:** Distribution of outdoor falls by focus group

**Location**	**Group no.**	***n***	**No. of outdoor falls**	***M*** **(SD) falls per participant**
Wales	1	6	11	1.83 (0.75)
	2	4	13	3.25 (0.96)
	3	6	11	1.83 (1.60)
England	4	5	5	1.25 (0.50)^1^
	5	7	17	2.43 (2.15)
	6	4	6	1.50 (1.00)
	7	3	6	2.00 (1.00)
Scotland	8	5	12	2.40 (1.67)
	9	4	7	3.50 (2.12)^2^
Total		44	88	

^1^Based on four fallers as one participant’s experience of falls did not meet our criteria and was excluded from the analysis.

^2^Based on two fallers as two participant’s experience of falls did not meet our criteria and was excluded from the analysis.

### Characteristics of outdoor falls: features of the physical environment

The frequencies of physical environmental characteristics of outdoor falls identified are summarised in Table 4. Outdoor falls occurred at different times of day and night and in all weather conditions, although in this sample outdoor falls appeared to be slightly more frequent in the winter and while walking on uneven pavements. Outdoor falls most frequently occurred when participants were in or crossing a road (22 out of 76 comments), usually when stepping up or down a kerb, crossing a road, or in three instances while getting out of a car:

“…I stepped off the kerb and as I was going off somebody said, ‘Come on, hurry up, the traffic is coming’, and I went down in front of the traffic…” (Group 2, JW1).

“…I was on [name] road coming to the [traffic] lights and across the lights there, the lights were for me you know? I crossed the road and there was a fella in a car come flying down right up to the lights and I went like this [raises her arms] and I stumbled and that was how it happened…” (Group 8, LR1).

**Table 4:** Characteristics of outdoor falls: Features of the physical environment

**Code**	**Subcode**	**Frequency of comments – no. of participants**^**1**^	**Frequency of comments - in total**^**2**^
Season			
	Spring	2	2
	Summer	4	4
	Autumn	3	3
	Winter	6	6
	*Total*	*15*	*15*
Time of day			
	Morning	4	4
	Afternoon	2	2
	Evening	2	2
	*Total*	*8*	*8*
Weather			
	Wet	5	6
	Cold	2	2
	Icy	1	1
	Warm	1	1
	Sunny	7	7
	*Total*	*16*	*17*
Lighting			
	Dark	4	5
Location			
	In/crossing road	18	22
	Car park	5	6
	Near home	9	11
	Garden	4	4
	Near shops	12	13
	Getting on a bus	1	1
	Getting off a bus	1	1
	Marina/promenade	2	3
	While on holiday	1	1
	Ambiguous	10	14
	*Total*	*63*	*76*
Footpath			
	Obstruction on footpath	2	2
	Pavement not flat	7	8
	Not on pavement	2	2
	Going down hill	2	2
	Climbing up hill	1	1
	Coming down steps	1	1
	*Total*	*15*	*16*

^1^At the participant level, i.e. each participant can only be counted once.

^2^At the overall level, i.e. each participant can be counted multiple times.

The other most frequent locations for outdoor falls were near shops (13/76 comments) or near to home (11/76 comments):

“I was on my own walking across the market square by the Guildhall and you know where the trees are, well I didn’t realise that the tarmac around the trees was so raised, you know what I mean, where the roots are sort of coming up, well, my foot caught on the tarmac and boy, did I go flying… of course as it was market day there were a lot of people around…” (Group 4, FB1).

“Falling out of the door was an embarrassment but we have got easy access now so it’s not a problem, but it was quite a nasty thing cos the pavement went like that and then you had a step and then you had the front door with a very high threshold and although I had a handle on the door, as I said my hands were full so I just went flying…” (Group 2, PB3).

### Characteristics of outdoor falls: features of the social environment

The frequencies of social environmental characteristics of outdoor falls identified are summarised in Table 5. In some cases it appeared that individual factors led to a higher risk of the fall occurring (e.g. the individual was rushing), and on occasion the individual was unable to get up unaided. Outdoor falls most frequently occurred when walking in a familiar area, marked as either a frequently walked route (17 out of 40 comments) or identified in discussion as a familiar route (18/40 comments). Of note is that data on familiarity can be identified from the features of the physical environment, in that several falls occurred near to home (11/76 comments), or in the garden (4/76 comments), with only one outdoor fall experienced while on holiday. In addition, outdoor falls occurred most frequently in the presence of other people, who often came to the aid of the participant (37/73 comments):

“So I was coming out of Lidls (supermarket), had my car there …I was just trying to open the back car door and my balance went and I was on the floor and a couple of people come along and helped me up” (Group 1, GM2).

**Table 5:** Characteristics of outdoor falls: Features of the social environment

**Code**	**Subcode**	**Frequency of comments – no. of participants**^**1**^	**Frequency of comments - in total**^**2**^
The individual			
	Rushing	5	5
	Not paying attention	4	5
	Carrying object(s)	4	4
	*Total*	*13*	*14*
Others			
	Alone	11	13
	In company	16	17
	With a dog	3	4
	People around	28	37
	Road traffic	2	2
	*Total*	*60*	*73*
Getting up			
	Couldn’t get up unaided	6	9
Familiarity			
	Frequent route	14	17
	Frequent venue	2	2
	Familiar area	16	18
	Not familiar	3	3
	*Total*	*35*	*40*

^1^At the participant level, i.e. each participant can only be counted once.

^2^At the overall level, i.e. each participant can be counted multiple times.

A similar number of outdoor falls took place when the individual was in familiar company (17/73 comments) and alone (13/73 comments):

“I went on the walk for health…and I was trotting along the person at the side of me and all of a sudden I feel as if I was flying through the air …” (Group 7, BB1).

“I had my fall walking along [name] street…really cracked my knee and it was so sore…trouble was that there was no one about to help me get up either, so I just sort of sat there too for while getting my breathing under control…” (Group 4, EP2).

### Attributions of outdoor falls

The frequency of attributions of outdoor falls are summarised in Table 6. A third of outdoor falls had no reported cause (29 falls) and in a fifth, the cause was reported as unknown (18 falls). Attribution of outdoor falls was rarely noted to be in relation to other people (3 falls) (e.g. someone pushing past and knocking them over). Instead, individuals reported that either they or some feature of their environment was to blame for their outdoor fall. Individual factors were mainly to do with poor health (7 out of 23 comments) or rushing (5/23 comments) at the time immediately preceding the fall:

“I was giving a lift to some friends and I was walking along in Swansea and I missed the pavement and that knocked my teeth out…my knee just gave way, I don’t think there was an issue with the pavement, they were walking with the same and they didn’t have a problem, it is just this sudden and my knee would give and down I go and I can’t predict it…” (Group 2, PB2).

“…I did have Christmas shopping in the trolley, big trolley and also a bag that I couldn’t get in the trolley in my right hand and things in my left hand and so I couldn’t stop myself from falling and then I saw the bus go round the corner and I decided in my wisdom that I would get that bus, I didn’t have to rush. I am always rushing, I can’t get out of the habit of rushing and the next minute, I only took two steps and got my foot caught in the trolley and hit the ground and I cracked my jaw …” (Group 5, FE3).

**Table 6:** Attributions of outdoor falls

**Code**	**Subcode**	**Frequency of comments – no. of participants**^**1**^	**Frequency of comments - in total**^**2**^
	Unknown	15	18
The individual			
	Health condition	5	7
	Rushing	5	5
	Symptoms at the time	3	3
	Shoes	2	3
	Misperception	2	2
	Unfamiliarity	1	1
	Lack of concentration	1	2
	*Total*	*19*	*23*
Other people			
	Person with them	2	2
	Passer by	1	1
	*Total*	*3*	*3*
Environment			
	Tripping hazard	3	3
	Uneven/poorly maintained pavement	6	7
	Dog – pulling/running into them	2	2
	Weather – slippery conditions	1	2
	Loud noise	1	1
	*Total*	*13*	*15*

^1^At the participant level, i.e. each participant can only be counted once.

^2^At the overall level, i.e. each participant can be counted multiple times.

The predominant environmental factor resulting in falls was uneven or poorly maintained pavements (7/15 comments):

“…when I had a fall in [road name]…and if you look down and it’s not those big pavings its those little square ones and it’s like the waves of the sea, it is exactly like that, and I said do you think that right?” (Group 5, NB7).

### Impact of outdoor falls

The frequency of comments in regard to the impact of outdoor falls are summarised in Table 7, in relation to both physical injury and emotional response.

**Table 7:** Impact of outdoor falls

**Code**	**Subcode**	**Frequency of comments – no. of participants**^**1**^	**Frequency of comments - in total**^**2**^
Injury			
	No injury	6	7
	Untreated injury	14	17
	Treated injury	16	17
	Fractures	10	10
	*Total*	*46*	*51*
Emotional response			
	Embarrassed	12	17
	Upset	7	7
	Angry	1	1
	Stunned	1	1
	Anxious of falling	17	20
	Not anxious of falling	2	2
	*Total*	*40*	*48*

^1^At the participant level, i.e. each participant can only be counted once.

^2^At the overall level, i.e. each participant can be counted multiple times.

#### Physical injury

Outdoor falls most frequently resulted in some injury. Untreated injuries included cuts and bruises such as black eyes, cuts to hands and on the head, twisted ankle, and concussion. More severe injuries requiring medical attention included deep cuts to a hand, a dislocated shoulder, displaced teeth, and cracked jaw. Fractures included breakages to the femur, ribs, wrist, arm, several fingers, and nose. Comparisons were made with outdoor falls that resulted in fractures with the dominant codes that emerged from the above analysis and which had most readily available data, namely setting (urban vs. rural), frequency of going out into their neighbourhood in a typical week (split at the median; up to 5 vs. 6 or more times), location of falls (in or crossing the road, near shops, and near home), presence of others (people around, in company, and alone), and attribution (unknown, poor health, rushing, and uneven pavements). Of the 10 outdoor falls that resulted in fracture, a strong pattern did not emerge as to the context in which they occurred as they occurred in urban (5) and rural settings (5), among those that had relatively fewer (6) or greater (4) number of outings in a typical week, occurred in or crossing a road (3), near to home (3), with people around (6), in company (4), alone (4), and with attributions that were unknown (4), due to poor health (1), and rushing (1),

#### Emotional response

A range of emotions were elicited in relation to outdoor falls, but most frequently embarrassment (17 out of 48 comments) and feeling upset (7/48 comments):

“I had just got off the bus to go across at the crossing to meet my daughter, she had gone to have her hair done and I just, I fell actually over nothing and ended up on the floor and really there was…just on the floor and I thought you know what a fool…” (Group 6, WW1).

“A flag was up and the sewer was slightly up as well and I just caught my…er…front of my shoe and over I went…and then looking at the shock, I had tights that were all torn and I was bleeding, my elbow was bleeding, sort of knocked my chin, so I thought ‘What shall I do?’ so I just sort of gathered my stuff…didn’t cry or anything but you sort of feel a bit emotional when you do something like that…” (Group 7, JH1).

It was also apparent that several participants were anxious about falling over again (20/48 comments):

“I tripped, I caught my foot in a really high tile and I went flat and I had just had well it was a year after I had my knee replacement. They said I didn’t do any damage to it, they x-rayed it and everything, but I have never been able to walk properly since and that’s 2007 and I am frightened, I am alright if I have got the wheels, I can go anywhere with my wheels but I am alright if I can feel something either side of me or one side at least so I am alright indoors cos I can mark my hallways and everything so I can hold on but the point is I can’t, if I am in the middle of a road I just freeze I can’t go backwards or forwards I am just stuck there unless I have got somebody with me and they say it’s all in the mind…..We did say it psychologically is one of the worse that is the thing that you never seem to get over do you?” (Group 5, JP2.1).

Comparisons were made with outdoor falls that resulted in anxiety about falling again with the dominant codes that emerged from the above analysis and which had most readily available data, namely setting (urban vs. rural), frequency of going out into their neighbourhood in a typical week (split at the median; up to 5 vs. 6 or more times), location of falls (in or crossing the road, near shops, and near home), presence of others (people around, in company, and alone), attribution (unknown, poor health, rushing, and uneven pavements), and physical injury (fractures). Of the 20 outdoor falls that resulted in anxiety about falling again, a strong pattern did not emerge as to the context in which they occurred as they occurred in or crossing a road (5), near shops (2), near to home (3), with people around (10), in company (4), alone (4), with attributions that were unknown (2), due to poor health (2), rushing (2), and uneven pavements (1), and that resulted in fracture (5). However, it did appear that anxiety about falling again was more prevalent among those that fell in urban (16) rather than rural settings (4), and among those that went out into their neighbourhood relatively more frequently in a typical week (14) than those who made fewer outings (6).

## Discussion

We conducted nine focus groups with older people across the UK to obtain accounts of their experiences of falls in outdoor environments, with the aims of exploring the self-reported contextual factors associated with outdoor falls and their interrelationship with injury and fear of falling. Using framework and content analyses, it appeared that our sample experienced outdoor falls in a variety of circumstances and with a range of resultant injury and emotional responses. Most frequently, it appeared that outdoor falls occurred when crossing roads, in a familiar area such as near to shops or home, and in the presence of bystanders. Half of the outdoor falls had either no reported attribution or an unknown cause. Of the attributions provided, they most frequently centred on poor health, rushing, and uneven or poorly maintained pavements. Reports of falls were most frequently accompanied with reports of injury that was either minor (e.g. bruising) or major (e.g. fractured femur), feeling embarrassed at the time, and anxiety about falling over again. While no strong pattern emerged as to the contexts of outdoor falls and their impact in terms of physical injury, anxiety about falling again appeared to be more prevalent among those who fell in urban settings and who made more outings into their neighbourhood in a typical week.

### Characteristics, attributions, and impacts of outdoor falls

Previously, outdoor falls have been identified as occurring more often on pavements, kerbs, and streets [12], and similarly, falls in our sample most frequently occurred while in or crossing a road. Stopping times at crossings in the UK have been suggested to be too short in duration for some older people to cross safely [32], and difficulty with short crossing times are associated with reduced outdoor physical activity among older people [33,34]. A study that changed outdoor features found women in particular appreciated longer road crossing times, and rollator users and those in better health appreciated greater separation between cyclists and pedestrians [35]. However, our sample did not mention short crossing times as an issue contributing to their outdoor falls and there are several other potential explanations. For example, it may be that an individual is inadequately able to perceive an obstacle such as a dropped kerb due to either low vision (such as poor depth perception) [25,36] or an inability to dual task (e.g. combining foot placement with watching for road traffic) [37,38], exacerbated by fatigue from the increased cognitive effort of going into outdoor spaces (e.g. due to unfamiliar noise) [39]. Future research is needed to ascertain the key risk factors for outdoor falls among subgroups of the older population, given the complexity of the interaction between the environment and older people’s health and physical activity [40-48] and low levels of physical activity among older people [49]. Road safety warrants particular attention as perceptions of road traffic safety influence walking behaviour in the general population [50] and mobility among older adults [51]. Indeed, noisy traffic, along with fear of moving outdoors and hills in the nearby environment have predicted self-perceived inadequate levels of physical activity among older people [52]. Density of road traffic, along with more difficult terrain and walking distances, has also predicted fear of moving outdoors among older people, which in turn predicts difficulty in walking and lower perceived quality of life [53,54]. Further research could identify hotspots for falls [17,36,55,56] and assess if the locations are associated with road safety.

Our study suggests that outdoor falls occur most frequently in a familiar area such as near to shops or home. This may reflect exposure to risk, as outdoor falls have been found to often occur while walking [13,36,57], and people afraid of falls - and particularly older women - are more likely to restrict their outdoor physical activity to familiar environments [58-60]. Mobility is also likely to be pertinent, given that older people’s walking speed and mobility declines with age and there can be several barriers to mobility in the built environment for people with mobility disabilities [61-63]. Exposure to falls risk may also reflect the local environment of the older person, given that closer proximity to services and amenities has been associated with frequent walking [64]. Indeed, close proximity to commercial destinations that facilitate social interaction (e.g. restaurants) or incidental social contact (e.g. hairdressers) have been found to promote walking among older people [65]. However, other studies have found that outdoor falls occurred more frequently in unfamiliar contexts [16,66], perhaps because participants were paying more attention to navigating their route than to potential fall hazards.

Outdoor falls in the current study occurred most frequently in the presence of bystanders. This proved useful as most bystanders came to the faller’s assistance, of which some were unable to get up unaided. However, several outdoor falls were associated with feeling embarrassed at the time of the fall and anxiety about falling over again in the future. It is possible that the presence of bystanders may intensify feelings of embarrassment at the time of an outdoor fall, which in turn intensifies anxiety about falling over again. Indeed, previous work has indicated that older people can fear the social consequences of falls - damage to pride, identity, embarrassment - more than physical injury [67].

It is of note that half the reported outdoor falls had either no reported attribution or an unknown cause. Most outdoor falls in a prior study had a known cause [36], and so our finding may reflect an inclination among participants not to offer causes for their falls in a group context due to fear of negative evaluation among peers [68,69]. The attributions provided centred on poor health, rushing, and uneven or poorly maintained pavements. These attributions resonate with risk factors for outdoor falls identified from previous studies: falls history, visual impairment, symptoms of depression [10], rushing [36], and uneven surfaces [12,35,36].

Outdoor falls resulted most frequently in an injury (whether treated or untreated), feeling embarrassed, and feeling anxious about falling over again. No strong pattern emerged as to the contexts of outdoor falls and their impact in terms of physical injury. A previous study found similar proportions of injury from outdoor falls with 68% of fallers experiencing minor injury [36]. However, most fatal falls have been found to occur indoors (75%) as they are more likely to occur amongst the oldest old [15], and most hip fractures have been found to occur indoors (83%) as they are more likely to occur among the frail community-dwelling and residents of long-term care institutions [19]. Similarly, mortality risk has been found to be elevated among those who have experienced an indoor but not an outdoor fall [20]. However, other studies have suggested that outdoor falls are just as likely as indoor falls to result in serious injury [11,17,70]. In regard to fear of falls, in our study it appeared that anxiety about falling again was more prevalent among those who fell in urban settings and those who made more outings into their neighbourhood in a typical week. Again, this may reflect exposure to risk of falls, given that while there was an equal spread of urban and rural participants who went out into their neighbourhood up to five times (11 and 10 respectively), more participants from urban settings frequented their neighbourhood six times or more compared with those from rural settings (16 and 3 respectively). Indeed, the only emergent pattern as to the context of outdoor falls in urban settings and among those with higher frequency of neighbourhood outings was the presence of bystanders and falls when in or crossing roads, which were more frequently reported by the overall sample. Further research could aim to replicate our findings while controlling for exposure to risk of falls.

### Limitations of the current study and suggestions for future research

The findings from this exploratory qualitative study suggest that further investigation is warranted regarding patterns of outdoor falls among older people. In particular, road safety emerged as an area of focus that may be key to the prevention of outdoor falls. Our other findings are less clear in relation to previous literature and future studies could provide further clarification around the following characteristics of outdoor falls: familiarity of surroundings, presence of bystanders, perceived attribution of falls, and their relation to the impact of falls. Given the study design, we cannot rule out the possibility of bias in the reports among participants in this study. As with other study designs that use self-reports, the participants in this study may have volunteered information and presented it in a manner to appear socially desirable. In addition, given the reports of outdoor falls were retrospective, there may well have been under-reporting of falls and in particular among recurrent fallers [71,72], and some details may have been missed in the accounts of the outdoor falls. Nonetheless, this exploratory study provides novel data that reflect the reporting of outdoor falls from older people’s perspectives to be followed up in future prospective studies that use objective measures where possible (e.g. medical records if investigating injurious falls).

We were successful in recruiting a heterogeneous sample in terms of age, health status, and location across the UK. However, we experienced difficulty in recruiting men to the focus groups. Future studies could have a special focus on older men given that they are more likely to experience outdoor falls [11,55], and interviews rather than focus groups may attract greater participation from men. In addition, further research could focus on older people from ethnic minorities, as all our participants were White British, and previous studies have reported on the role of culture on attitudes toward falls [73]. Finally, further studies are warranted that recruit participants across all seasons, as prior studies have clearly identified a higher number of falls during winter [55,74]. While our study conducted in spring/summer tentatively suggested a higher frequency of outdoor falls during winter, further work is needed to confirm seasonal effects and to ascertain if people’s perception of outdoor falls risk alters across seasons.

## Conclusions

This exploratory study has highlighted several aspects of the outdoor environment that may pose as risk factors for future falls. Our findings suggest that future research should focus on the risks of falling in familiar areas such as streets near to home and in particular when crossing roads, and to effective strategies to reduce fear of falls among people in urban settings and who frequently make outings into their neighbourhood. Health professionals who work to prevent falls and increase mobility among older people are recommended to consider the reduction of falls and fear of falling in outdoor environments as well as within the home.

## Competing interests

The authors declare that they have no competing interests.

## Authors’ contributions

SRN: Lead for the project; led on conception and design, supervised researcher for data collection, led on analysis and interpretation, wrote first draft of manuscript. CB: Contributed to design, analysis and interpretation, and critical revision of manuscript drafts. JEP: Contributed to design, data collection, interpretation of analysis, and critical revision of manuscript drafts. RN: Contributed to conception and design, data collection, interpretation of analysis, and critical revision of manuscript drafts. All authors read and approved the final manuscript.

## Pre-publication history

The pre-publication history for this paper can be accessed here:

http://www.biomedcentral.com/1471-2318/13/125/prepub

## Supplementary Material

Additional file 1 **Focus group schedule.** Schedule used by the focus group facilitator including semi-structured focus group questions. Click here for file
