# Supplementary material for: Characteristics of outdoor falls among older people: a qualitative study
Source: BMC Geriatr. 2013 Nov 18;13:125. doi: 10.1186/1471-2318-13-125 (PMC3835551; doi:10.1186/1471-2318-13-125)
Supplement: Additional file 1 — Focus group schedule. Schedule used by the focus group facilitator including semi-structured focus group questions. [file 1471-2318-13-125-S1.docx]

**Additional file 1: Focus group schedule**

The aim of the session is to elicit the participants’ views and experiences of outdoor falls and how they might be prevented. Focus groups are different to interviews in that the aim of the session is for the facilitator to afford participants to interact with each other. The facilitator’s role will be to keep the dialogue moving, to encourage quieter members to contribute, and ensure each participant is given opportunity to speak.

Research question

What are the experiences and understandings of older people about risk factors for falling in the outdoor environment?

*Subquestions:*

- What are older people’s experiences of falling outdoors?
- What are the characteristics of the outdoor falls they describe? (E.g. Where? When? Alone/in company? Familiar/unfamiliar spaces? Activity at time of fall? Weather? Light? Trajectory of fall? What occurred after the fall?)
- What do older people identify as the risk factors for outdoor falls?
- How does the social context and familiarity of the environment contribute to outdoor falls?
- How could these risk factors be modified? How might this be achieved?
- To what extent do older people feel empowered to effect change necessary to reduce risk of outdoor falls?

What follows is an outline of the questions that will be asked during the approximate <90 minute session. However, this is an outline and the interviewer may deviate from the schedule and develop the questioning if it exposes information that may benefit the research study.

Introduction

- Purpose of project to get your views and experiences of outdoor falls
- Ground rules for focus groups (in particular confidentiality)
- Format of focus groups
- Valuing your ideas and comments – YOU ARE THE EXPERTS!
- Reiterate consent procedures, and opportunity to ask questions
- Introductions
- Obtain informed consent

Topics for discussion (not necessarily in this order or exhaustive):

1. Can you talk about your experiences of having a fall outside?

Prompt: Near falls, slips, trips…?

Prompt: Can you talk about the most recent time you fell outdoors?

Prompt: Can you talk about what happened?

1. Can you talk about what you remember doing before you fell?

Prompt: Where were you, who were you with, what were you doing?

Prompt: What time of day was it?

Prompt: Were you somewhere familiar / unfamiliar?

Prompt: What was the weather like? Was it dark?

1. Can you talk about what happened when you fell?

Prompt: Did you fall backwards, forwards, or….?

Prompt: Were you hurt?

Prompt: Was anyone around to help you / check you were alright?

Prompt: Was the fall something you’ve thought about much since?

- 1. What different things do you think led to the fall happening?

Prompt: Person themselves, or others around them, lighting, busy place, etc.?

1. What kinds of things do you think need to be done in order to help prevent outdoor falls?

Prompt: What could the council be doing in (location) to prevent falls?

Prompt: What would make places around (location) safer?

Prompt: If the council could change one or two things to make outdoor falls less likely, what do you think they should do?

1. Do you think there is anything that older people, or those that are out and about with older people, could do to prevent falls outside?

Prompt: Is there anything that can be done?

Prompt: What do you feel confident about doing that might reduce the chances of having a fall?

Conclusion

- Anyone want to add anything else?
- Thank participants for their contributions
- Ask participants to complete the questionnaire
- Remind participants of the ground rules such as confidentiality.
- If you want to talk on individual basis, facilitators will be around afterwards
- We would like to send you a summary of our findings. Where to?
- Provide debrief forms – note our contact details
- Make arrangements for their travel home, and reimbursement of expenses
